# Expression and Significance of TRIM 28 in Squamous Carcinoma of Esophagus

**DOI:** 10.1007/s12253-018-0558-6

**Published:** 2018-11-27

**Authors:** Bo Liu, Xiujuan Li, Fengxi Liu, Fengyu Li, Shuxia Wei, Junchao Liu, Yang Lv

**Affiliations:** 1 Department of Pathology, First Affiliated Hospital of Hebei North University, Zhangjiakou, 075000 China; 2 Department of Histology and Embryology, Hebei North University, 11 Zuanshi South Road, Zhangjiakou, 075000 Hebei China; 3 Department of Oncology, No.251 Hospital of the Chinese People’s Liberation Army, Zhangjiakou, 075000 China

**Keywords:** Esophageal squamous cell carcinoma, TRIM28, Immunohistochemistry, Western blot, Immunofluorescence, Overall survival

## Abstract

Tripartite motif-containing protein 28 (TRIM28) has been proved to accelerate cell proliferation and metastasis in a variety of human cancers. However, the role of TRIM28 in esophageal squamous cell carcinoma (ESCC) remains unclear. In this study, to compare the biological effect and significance of TRIM28 expression in ESCC, immunohistochemistry (streptavidin-perosidase, S-P) method was used firstly to examine the expression of TRIM28 in 136 cases of ESCC, 35 cases of high grade intraepithelial neoplasia (HGIN), 29 cases of low grade intraepithelial neoplasia (LGIN) and 37 cases of normal esophageal epithelium (NEE). Then the associations of TRIM28 expression with clinicopathological data and overall survival (OS) were also analyzed. Western blot was performed to evaluate TRIM28 protein in a total of 20 matched human ESCC and NEE tissues. Moreover, the localization of TRIM28 protein in ESCC and NEE tissues was also detected by immunofluorescence. TRIM28 protein was mainly distributed in the nucleus of ESCC. The expression of TRIM28 increased progressively from NEE to LGIN, to HGIN, and to ESCC, and it was also related to invasive depth, pTNM stage and lymph node metastasis in ESCC (*P* < 0.05). The results of western blot and immunofluorescence all showed that the relative expression of TRIM28 protein was markedly upregulated in ESCC compared with the NEE tissues (*P* < 0.01). However, prognostic analysis showed that TRIM28 may not be a prognostic factor of patients with ESCC. In conclusion, the overexpression of TRIM28 may play an important role for development and metastasis in ESCC.

## Introduction

Esophageal carcinoma is the eighth most prevalent cancer and the seventh leading cause of cancer-related death worldwide [1]. And moreover, the morbidity and mortality of esophageal carcinoma in China accounted for the fifth and fourth of malignancy, respectively [2]. Esophageal squamous cell carcinoma (ESCC) is the most common histological type of esophageal carcinoma in China. With the progress in molecular biology and the increased level of cancer treatment, the 5-year overall survival (OS) rate of ESCC patients after esophagectomy has significantly increased over the past several decades but remains approximately 20% [3–6]. However, so far the molecular mechanism of ESCC genesis and progression is not well understood, and biomarkers for predicting clinical outcome of ESCC patients are also unavailable. Therefore, there is an urgent need to find sensitive and specific biomarkers for ESCC.

Tripartite motif (TRIM) family proteins, which contain a Really Interesting New Gene finger domain (RING), B-box zinc finger domain and a coiled coil region, are considered important regulators of carcinogenesis [7]. TRIM28, also known as transcription intermediary factor 1(TIF1β) or Krüppel associated box (KRAB)-associated protein 1(KAP1), is a universal co-repressor for a family of KRAB domain containing zinc finger proteins (KRAB-ZFPs), which constitute the single largest group of transcriptional repressors encoded by the genomes of higher organisms [8]. As one of the evolutionarily conserved TRIM family proteins, TRIM28 has been proved to accelerate cell proliferation and metastasis in a variety of human cancers. Studies have shown, for example, that TRIM28 knockdown may be effective against NSCLC, and the knockdown of TRIM28 expression by lenti-siRNA/TRIM28 may inhibit tumor growth and induce cell apoptosis in vivo [9, 10]. Hao L found that TRIM28 is frequently elevated in multiple tumor types and is associated with aggressive clinical features of breast cancer. Furthermore, the overexpression of TRIM28 was significantly correlated with poor prognosis in patients with breast cancer [11]. In glioblastoma and liver cancer, TRIM28 is also involved in a wide range of biological processes and its over-expression is associated with poor outcome in patients [12, 13]. However, the role of it in ESCC is still not clear. In this study, we investigated the expression pattern of TRIM28 in ESCC, and evaluated its relationship with clinicopathological features and survival.

## Material and Methods

### Patients and Samples

As described in detail previously [14], all patients were treated at the No.1 Affiliated Hospital of Hebei North University for ESCC from January 1, 2008 to December 31, 2009 and those with complete clinical data were enrolled. All procedures involving human participants were performed in accordance with the ethical standards of the institutional and/or national research committee and with the Helsinki Declaration and its later amendments or comparable ethical standards. A total of 136 ESCC patients were included in the present study and they were diagnosed based on pathological findings. These patients were 91 men and 45 women, ranging in age from 27 to 80 years (mean ± SD: 61.2 ± 8.9 years). The tumor-node-metastasis (TNM) stage of the ESCC patients was defined according to the 7th edition of the UICC-AJCC TNM staging system [15]. For 35 cases of high grade intraepithelial neoplasia (HGIN) patients, they were 24 men and 11 women, ranging in age from 34 to 78 years (mean ± SD: 57.8 ± 8.1 years). For 29 cases of low grade intraepithelial neoplasia (LGIN) patients, they were 19 men and 10 women, ranging in age from 32 to 72 years (mean ± SD: 55.9 ± 7.8 years). For 37 cases of normal esophageal epithelium (NEE), they were 20 men and 17 women, ranging in age from 43 to 71 years (mean ± SD: 59.5 ± 8.0 years). Statistics showed that the general data were not different among the 4 groups. Informed consent was obtained from all individual participants included in the study.

### Follow up

For 136 cases of ESCC patients, they were followed up from operation time to December 31, 2016 by telephone and return visits, with an interval of 3 months, and death was considered an event. Post-operative metastasis and recurrence were diagnosed on the basis of clinical examination, imaging assessment, operative and pathologic examination. Clinicopathologic data were obtained from pathologic reports, laboratory examination, medical records, and imaging, primarily including information of gender, age, tumor location, tumor size, histological differentiation, invasive depth, pathological TNM (pTNM) stage, lymph node metastasis, postoperative radiotherapy and chemotherapy, as described in detail previously [14] .

### Immunohistochemistry (IHC)

The appropriate paraffin-embedded specimen blocks of each case were obtained from the Department of Pathology. Tissue sections (4 μm thick) were dried at 60 °C for 3–4 h, deparaffinized with three 10-min washes in xylene, and rehydrated in decreasing concentrations of ethanol in distilled water. Next, the sections were soaked in boiling sodium citrate buffer (ZLI-9065, 0.01 M, pH = 6.0, Zhongshan Goldenbridge Biol\Technology Co., Ltd., Beijing, China) for 20 min in microwave oven. When cooled to room temperature (RT), the sections were washed 5 min in phosphate buffer solution (PBS, ZLI-9063, 0.01 M pH 7.2–7.4, Zhongshan Goldenbridge Biol\Technology Co., Ltd., Beijing, China) for three times. Then, the tissue sections were treated with 3% hydrogen peroxide for 10 min to block endogenous peroxidase activity. After washing 5 min in PBS for three times, the sections were incubated overnight at 4 °C with rabbit polyclonal anti-TRIM28 antibody (GTX102226; dilution, 1:200; GeneTex, USA) and followed by incubation with reagent I of HRP-labeled goat anti-rabbit IgG antibody (SP-9001, Zhongshan Goldenbridge Biol\Technology Co., Ltd., Beijing, China) for 30 min at 37 °C. The sections were washed 5 min in PBS for three times. After incubating with reagent II of HRP-labeled goat anti-rabbit IgG antibody (SP-9001, Zhongshan Goldenbridge Biol\Technology Co., Ltd., Beijing, China) at 37 °C for 30 min and washing with PBS for 5 min (three times), sections were counterstained with 5% hematoxylin (H8070, Beijing Solarbio Technology Co., Ltd. Beijing, China) for 5 min. Finally, the sections were blued in 1% hydrochloric-acid alcohol, dehydrated in increasing concentrations of ethanol, cleared with xylene, and mounted in neutral gum under a coverslip. The sections treated without primary antibody (use PBS as a substitute) were used as negative control.

### Evaluation of Immunohistochemistry

Using a high-power (400×) microscope (BX53, Olympus, Japan), TRIM28 expression as evaluated by two experienced pathologists independently, without knowledge of the clinical information. For each slide, five random non-overlapping fields containing at least 200 cells per field were observed and scored based on the percentage of positively stained cells (score 0 for negative, 1 for <10%, 2 for 10–50%, 3 for 51–80%, 4 for >80%) and the staining intensity (score 0 for negative staining, 1 for weak staining, 2 for moderate staining, and 3 for strong staining).

The intensity and proportion scores were multiplied to generate the IHC index. The expression level was considered as low (IHC index<6), and as high (IHC index≥6) [16].

### Western Blot

A total of 20 matched human ESCC tumor tissues and adjacent NEE tissues were collected directly after surgical resection was performed at the No.1 Affiliated Hospital of Hebei North University (China). All of the tissue samples from patients with no prior neoadjuvant treatment were immediately frozen in liquid nitrogen and stored at −80 °C until protein was extracted. Clinicopathological information for all of the samples was available. Our research protocol was approved by the Ethics Review Committee of the Institutional Review Board of the hospital. Standard written consent was obtained from each patient. Western blot analyses were performed on the 20 matched human ESCC and NEE tissues. The main experimental steps were as follows: Cells were washed with PBS which had been precooled at 4 °C firstly. Then lysis buffer (#9803, 10×, CST, USA) was added and centrifuged to save the supernatant containing proteins. The proteins were separated by SDS-PAGE (P0012AC, Beijing Beyotime Technology Co., Ltd. Beijing, China), and then transferred to polyvinylidene fluoride (PVDF, FFP20,Beijing Beyotime Technology Co., Ltd. Beijing, China) membranes which had been pretreated with methanol. After that, the primary antibodies, anti-TRIM28 antibody (GTX102226; dilution, 1:5000; GeneTex, USA) antibody and anti-β-actin (sc-81,178; dilution,1:1000; Santa cruz, USA) antibody were added to each group, respectively. Next, the groups were incubated at 4 °C overnight. The membranes were washed with TBST (P0231, Beijing Beyotime Technology Co., Ltd. Beijing, China) for three times, and then incubated with goat anti-rabbit (sc-2004, 1:5000; Santa cruz, USA) antibody at 37 °C for 2 h. Western blot analysis was used by Image Lab 3.0 (BIO-RAD, USA). Then the grayscale of each blot was measured 3 times and normalized to α-tubulin.

### Immunofluorescence (IF)

Immunofluorescence analysis was employed to investigate the expression and localization of TRIM28 protein in ESCC and NEE. Tissue sections (4 mm thick) were dried at 60 °C for 3–4 h, deparaffinized with two 10-min washes in xylene, and rehydrated in decreasing concentrations of ethanol in distilled water. After endogenous peroxidase activity was quenched with 3% H_2_O_2_ for 15 min. After washing three times for 5 min in PBS, they were treated with 1% Triton X-100-PBS at RT for 20 min, and washed with PBS again, blocked with normal goat serum (ab7481, Abcam, UK) for 60 min. After that, the tissues were incubated with rabbit polyclonal anti-TRIM28 antibody (GTX102226; dilution, 1:200; GeneTex, USA) at 4 °C for 24 h. Next, they were stained with FITC-conjugated anti-Rabbit secondary antibody (Anti-rabbit IgG (h + l) Ab, Dl488 072–03–15-06, dilution, 1:50; KPL, USA) in the dark at RT for 60 min and washed with PBS three times. Then, we added 10 μL of 4, 6-diamidino-2-phenylindole (DAPI; D9542, Sigma, USA) counterstain into the area of the specimen. Finally, the cover slip was sealed with Fluorescent Mounting Media (S2100, Beijing Solarbio Technology Co., Ltd. Beijing, China). Negative controls were also employed to offset the disturbance of the primary or secondary antibody. The results were observed and recorded by fluorescence microscopy (Leica TCS-ST2 Instrument, Japan).

### Statistical Analysis

Statistical analysis was performed using the SPSS v.17.0 software (USA). Data were expressed as mean ± SD. The one-way ANOVAs were used to analyze the results of Western blot and immunofluorescence. Furthermore, the relationships of TRIM28 expression between ESCCs and noncancerous tissue, TRIM28 expression with the clinicopathologic parameters were all assessed using Chi-square and Fisher’s exact tests. Kaplan–Meier survival analysis and log-rank tests were used to evaluate the 5-year OS rates of ESCC patients. Cox univariate analysis was used to determine the prognostic significance of variables, and Cox multivariate analysis was applied to identify independent prognostic factors for ESCC. The one-way ANOVAs were used to Western blot analysis. For all results, differences were considered to be statistically significant at *P* < 0.05 with a 95% confidence interval.

## Results

### Association Between TRIM28 Expression and Clinicopathological Parameters of ESCC by IHC Analysis

**Fig. 1:**
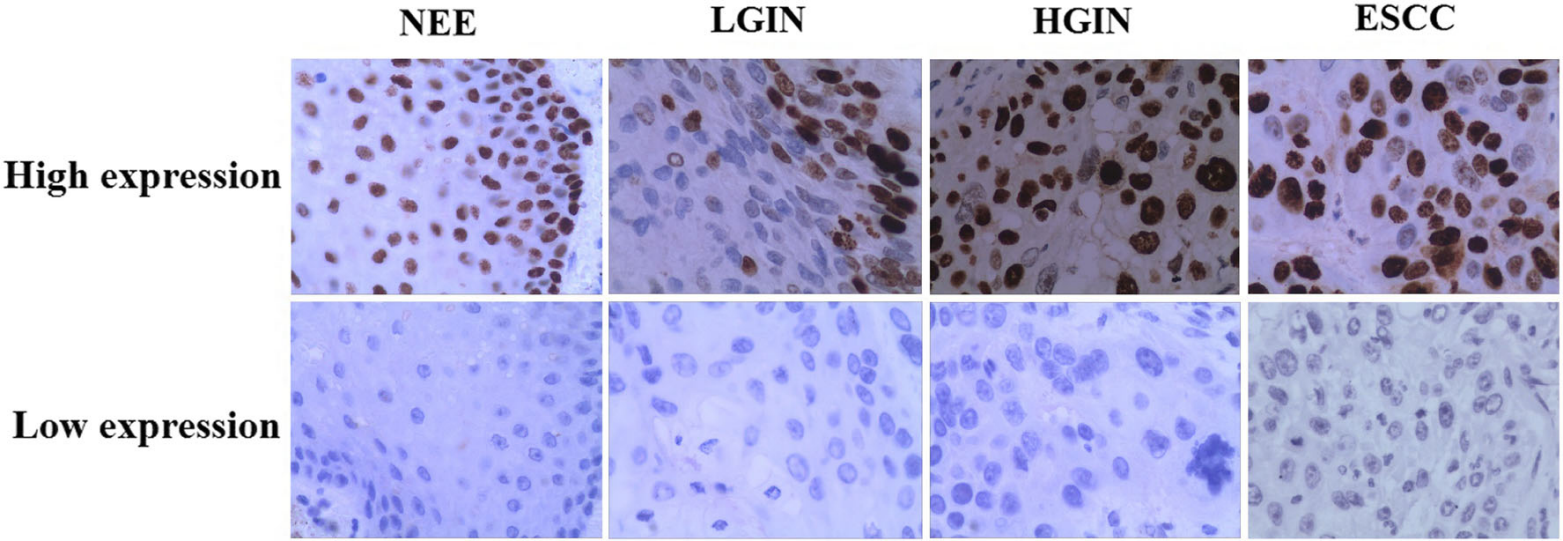
Representative immunohistochemical staining of TRIM28, ×400

**Fig. 2:**
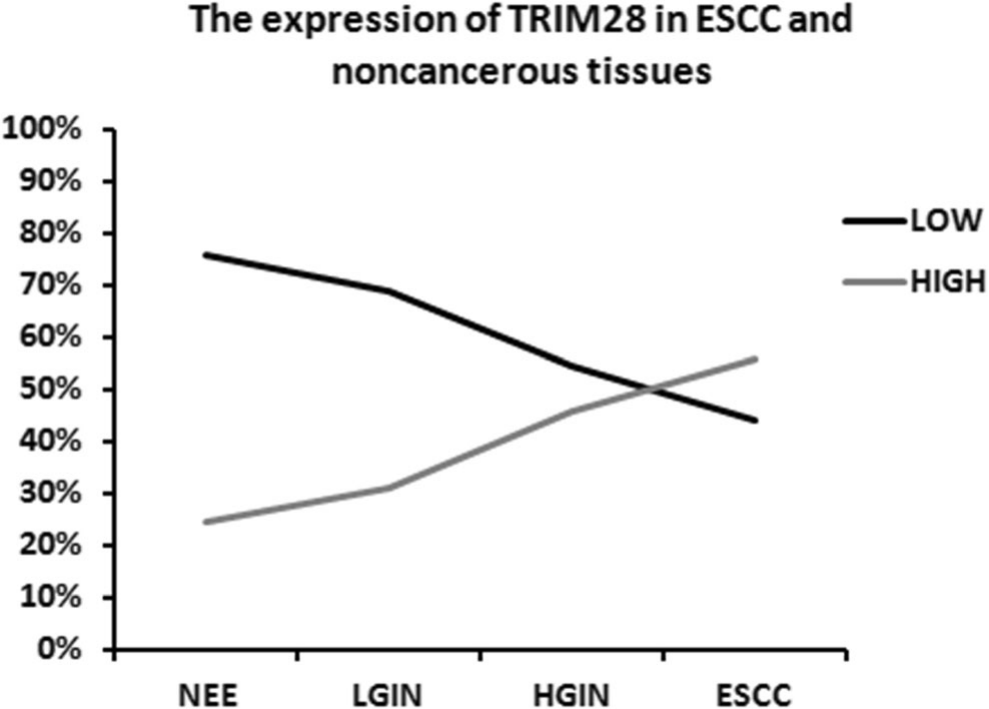
The expression of TRIM28 in ESCC and noncancerous tissues (Chi-square and Fisher’s exact tests)

TRIM28 was measured in NEE, LGIN, HGIN and ESCC. IHC analysis showed that the frequency of TRIM28 protein expression was lowest in NEE (24.3%) and increased gradually during the evolution of esophageal carcinogenesis, with 55.9% of ESCC showing high expression of TRIM28 protein (Table 1, Fig. 1). Furthermore, a χ^2^ test showed that there was a significant difference when comparing the prevalence of TRIM28 expression in various levels of cancer progression (χ^2^ = 14.926, *P* = 0.002) (Fig. 2). Moreover, the abnormal expression of TRIM28 was related to pTNM stage, invasive depth and lymph node metastasis (*P* < 0.05) (Table 2).

**Table 1:** TRIM28 protein expression during cancer progression by IHC analysis

Cancer progression	TRIM28 expression [cases (%)]		*P* value	
	Low	High		
NEE^①^	28(75.7)	9(24.3)	①:② *P* = 0.544	①:③ *P* = 0.057
LGIN^②^	20(69.0)	9(31.0)	①:④ *P* = 0.001	②:③ *P* = 0.231
HGIN^③^	19(54.3)	16(45.7)	②:④ *P* = 0.015	③:④ *P* = 0.282
ESCC^④^	60(44.1)	76(55.9)

**Table 2:** Associations between the expression of TRIM28 and clinicopathologic characteristics of the 136 cases of ESCC patients

Characteristic	TRIM28 expression [cases (%)]		*P* value
	Low	High	
Total	60	76	
Gender			0.248
Male	37(61.7)	54(71.1)	
Female	23(38.3)	22(28.9)	
Age (years)			0.648
*n* ≤ 30	0(0)	1(1.3)	
30 < *n* ≤ 60	29(48.3)	38(50.0)	
*n* > 60	31(51.7)	37(48.7)	
Histological differentiation			0.687
Well	19(31.7)	20(26.3)	
Moderate	34(56.7)	44(57.9)	
Poor	7(11.7)	12(15.8)	
Invasive depth			0.000
T1–2	10(16.7)	2(2.6)	
T3	26(43.3)	17(22.4)	
T4	24(40.0)	57(75.0)	
pTNM stage			0.000
I	10(16.7)	1(1.3)	
II	26(43.3)	16(21.1)	
III	24(40.0)	43(56.6)	
IV	0(0)	16(21.1)	
Lymph node metastasis			0.004
Absence	46(76.7)	40(52.6)	
Presence	14(23.3)	36(47.4)	

### Relationships Between TRIM28 Expression and Survival of the Patients

The association between TRIM28 protein expression and OS of ESCC was estimated using log-rank test and multivariable Cox proportional hazard regression analysis (Table 3). The 5-year OS rate for all 136 ESCC patients was 13.2%. Kaplan-Meier survival analysis showed that the 5-year OS rate was significantly correlated with histological grade, pTNM, invasion depth, lymph node metastasis. However, the results of statistical analysis showed that TRIM28 may not be a prognostic factor of patients with ESCC. In addition, no LGIN became ESCC during follow-up period. But in 35 cases of HGIN patients, 16 cases of them became ESCC. Kaplan-Meier survival analysis also showed that TRIM28 was not a prognostic factor of patients with HGIN. Multivariate analysis demonstrated that histological grade, pTNM and invasion depth were all significant prognostic factors for 5-year OS rate of patients with ESCC.

**Table 3:** Associations between the expression of TRIM28, clinicopathological parameters and 5-year overall survival rate of the 136 cases of ESCC patients

Variables	Subset	HR	95% CI	*P*
Univariate analysis				
Age	≤60 vs. >60	1.070	(0.713–1.604)	0.745
Gender	Male vs. Female	1.221	(0.808–1.844)	0.343
Histological grade	G1 vs. G2–G3	2.381	(1.402–4.403)	0.001
Invasion depth	T1 + T2 vs. T3 + T4	2.307	(1.371–3.879)	0.002
pTNM	No vs. Yes	1.733	(1.178–2.551)	0.005
Lymph node metastasis	I–II vs III–IV	1.732	(1.053–2.820)	0.030
TRIM28	Low vs. High	1.341	(0.803–2.239)	0.262
Multivariate analysis				
Histological grade	G1 vs. G2–G3	0.506	(0.301–0.853)	0.011
Invasion depth	T1 + T2 vs. T3 + T4	0.391	(0.185–0.827)	0.014
pTNM	I–II vs III–IV	2.407	(1.264–4.582)	0.007
Lymph node metastasis	No vs. Yes	0.770	(0.459–1.290)	0.320

### The Expression of TRIM28 Between ESCC and NEE by Western Blot

**Fig. 3:**
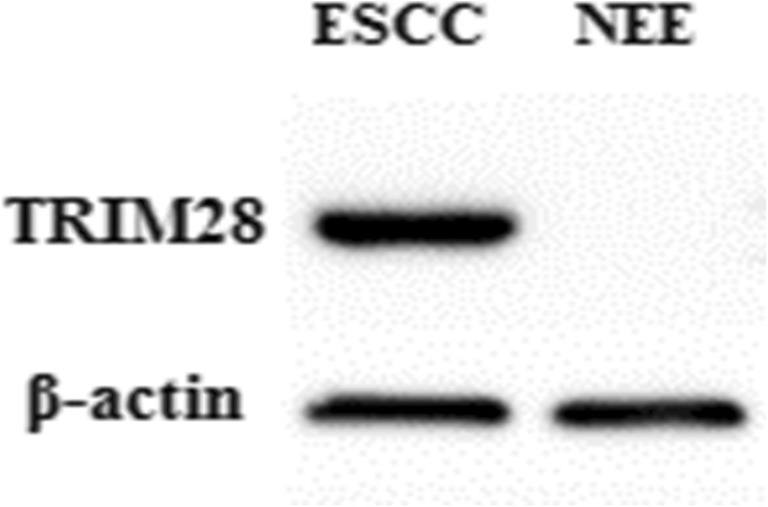
Relative expression of TRIM28 protein by Western blot between paired ESCC tissues and NEE tissues (Cells in ESCC can express TRIM28 while there was no expression of TRIM28 in cells of NEE)

The expression of TRIM28 was also detected by Western blot between a total of 20 matched human ESCC tumor tissues and adjacent NEE tissues. Our results showed that cells in ESCC can express TRIM28 while there was no expression of TRIM28 in cells of NEE (Fig. 3).

### Distribution of TRIM28 Protein in ESCC by IF

**Fig. 4:**
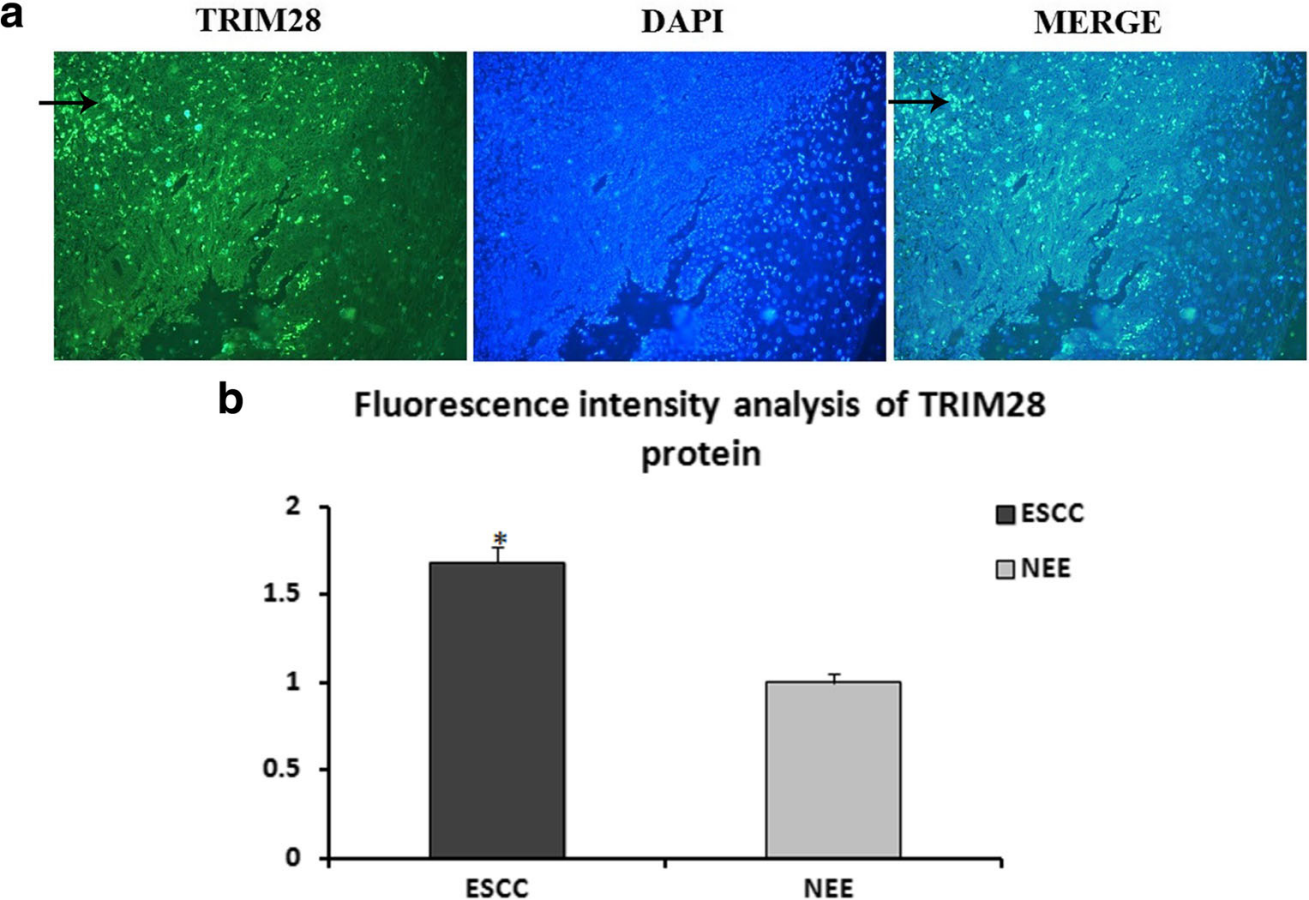
The distribution of TRIM28 protein in ESCC and NEE tissues by immunofluorescence stainings, ×400. (TRIM28 protein was stained with green fluorescence(→), which was expressed in the nucleus in ESCC cells and nuclei were counterstained with DAPI (A); Analysis results of the fluorescence intensity (B), *P* = 0.000) (One-way ANOVA)

Immunofluorescence staining was performed to show the distribution patterns of TRIM28 protein in ESCC and NEE. Under the confocal-microscope observation, TRIM28 protein was stained with green fluorescence, which was expressed in the nucleus of ESCC cells (Fig. 4A). Normal esophageal epithelial cells labeled by DAPI show blue under fluorescence microscope, which did not express TRIM28. Analysis of the fluorescence intensity revealed that the expression of TRIM28 in ESCC was noticeably higher than that of NEE (F = 1417.061, *P* = 0.000; Fig. 4B).

## Discussion

In the present study, we demonstrated that TRIM28 expression was significantly increased in ESCC and was associated with pTNM stage, invasive depth and lymph node metastasis.

Tripartite motif (TRIM) family proteins are a highly conserved group of E3 ubiquitin ligase proteins that can establish substrate specificity for the ubiquitin-proteasome complex and also have proteasome-independent functions [17]. They are considered important regulators of carcinogenesis and participate in many cellular processes, such as cell growth, development and cellular differentiation and alteration of them can affect transcriptional regulation, cell proliferation, autophagy and apoptosis [18, 19]. In recent years, many research studies have shown that TRIM family proteins, such as TRIM3 [20], TRIM11 [21], TRIM19 [22], TRIM31 [23], TRIM44 [24] and TRIM59 [25, 26] have been demonstrated to serve a significant role in the tumorigenesis and progression of numerous cancer types.

Among TRIM family proteins, tripartite motif-containing 28 (TRIM28), also known as KRAB domain-associated protein 1 (KAP1) or transcriptional intermediary factor 1 beta (TIF1β), is a large multi-domain protein (110 kDa), which is a member of a family of almost 60 human TRIM proteins. At the amino (N) terminus, TRIM28 protein contains four conserved structural domains that include a RING (Really Interesting New Gene) finger, two B-boxes, and a leucine zipper coiled-coil region (CC), which are collectively called the RBCC or TRIM domain [27].

To date, the clinical relevance of TRIM28 in diseases remains elusive. For example, Li F [28] compared TRIM28 expression between cervical cancer and adjacent normal tissues, and detected significant elevation in TRIM28 expression levels in the cervical cancer tissues. Their results also showed that the TRIM28-overexpressing tumors grew at a much higher rate, as determined by size and weight, than the control tumors, whereas the tumors formed by TRIM28-silenced cells were smaller and had lower tumor weights than those formed from shRNA-vector control cells. They believed that TRIM28 played a pivotal role in cervical cancer cell proliferation and might serve as a potential therapeutic target. In non-small cell lung cancer (NSCLC) cell lines, Liu L [9] demonstrated that the stable silencing of TRIM28 expression by a specific siRNA lentivirus vector significantly inhibited the growth and exerted obvious anti-tumor effects in nude mice. Furthermore, TRIM28 expression was significantly correlated with several clinicopathological characteristics of patients with breast cancer (BC), such as p53 mutation, tumor recurrence and Elston grade of the tumor [11]. TRIM28 overexpression was also detected in gastric cancer, ovarian cancer, glioma and hepatocellular carcinoma [12, 29–31], suggesting that TRIM28 upregulation is a common feature of many epithelial cancers. Research into the correlation between TRIM28 expression and prognosis in patients with cancer, Hao L [11] demonstrated that high expression of TRIM28 is a predictor of poor prognosis in patients with breast cancer. In addition, TRIM28 overexpression was also a predictor for poor prognosis of ovarian cancer [29], hepatocellular carcinoma [31] and thyroid carcinomas [32] patients.

However, there is no report on the correlation between TRIM28 and esophageal cancer. In this study, immunohistochemistry method was firstly used to examine the expression of TRIM28 in 136 cases of ESCC, 35 cases of HGIN, 29 cases of LGIN and 37 cases of NEE. TRIM28 protein was mainly distributed in the nucleus of ESCC. And similar to other epithelial cancers, the expression of TRIM28 increased progressively from NEE to LGIN, to HGIN, and to ESCC, and it was related to pTNM stage, invasive depth and lymph node metastasis in ESCC. Furthermore, Western blot and immunofluorescence results in our study also demonstrated that the relative expression of TRIM28 was markedly upregulated in ESCC samples compared with NEE tissues. The above results indicate that the abnormal expression of TRIM28 may play an important role for development and metastasis in ESCC. However, TRIM28 may not be a prognostic factor of patients with ESCC according to our results. And it was similar to the results of Chen L [33]. They revealed that TRIM28 overexpression is associated with better overall survival of patients with early-stage lung cancer, suggesting that TRIM28 may also have anti-proliferative activity to some tumor cells. The relevant mechanism also need further study.

## Conclusions

Taken together, the data provide that TRIM28 overexpression plays a role in development and metastasis in ESCC. Examination of TRIM28 may be useful for diagnosis and therapy in ESCC.
